# A cylindrical core-shell-like TiO_2 _nanotube array anode for flexible fiber-type dye-sensitized solar cells

**DOI:** 10.1186/1556-276X-6-94

**Published:** 2011-01-18

**Authors:** Jiefeng Yu, Dan Wang, Yining Huang, Xing Fan, Xin Tang, Cong Gao, Jianlong Li, Dechun Zou, Kai Wu

**Affiliations:** 1 BNLMS, College of Chemistry and Molecular Engineering, Peking University, Beijing 100871, China

## Abstract

A versatile anodization method was reported to anodize Ti wires into cylindrical core-shell-like and thermally crystallized TiO_2 _nanotube (TNT) arrays that can be directly used as the photoanodes for semi- and all-solid fiber-type dye-sensitized solar cells (F-DSSC). Both F-DSSCs showed higher power conversion efficiencies than or competitive to those of previously reported counterparts fabricated by depositing TiO_2 _particles onto flexible substrates. The substantial enhancement is presumably attributed to the reduction of grain boundaries and defects in the prepared TNT anodes, which may suppress the recombination of the generated electrons and holes, and accordingly lead to more efficient carrier-transfer channels.

## Introduction

Conventional flexible fiber-type dye-sensitized solar cells (F-DSSCs) based on polymer/ITO (indium tin oxides) usually suffer from several problems such as cost inefficiency, stringent temperature restriction, and light-reception-angle limitation. Recent advances in fiber- and mesh-type DSSCs that can be woven into a variety of shapes and forms provide a potential solution to above problems [1-3]. TiO_2 _electrodes can be fabricated by depositing a layer of disordered TiO_2 _particles on flexible substrates or fibers. However, this method can lead to twisted carrier-transfer channels that thereafter lower the efficiencies. This disadvantage can be presumably overcome by employing a fiber-like anode with a hierarchical crystalline TiO_2 _nanostructure, which may reduce the grain boundaries and defects, and thus leads to more efficient carrier-transfer channels.

To achieve this goal, it is necessary to design a novel fiber-type anode that possesses a hierarchical crystalline TiO_2 _structure to reduce the grain boundaries and defects, and maintains a relatively high surface area in the meanwhile. Electrochemical anodization can be used to anodize a Ti wire into a cylindrically core-shell-like TiO_2 _nanotube (TNT) array anode. In particular, this anodization process can greatly simplify anode post-processing by employing un-anodized inner Ti cores as the electric conduction leads. Electrochemical anodization has been widely employed to anodize metals into porous oxide membranes, such as anodic aluminum oxide (AAO) [4] and anodic titanium oxide (ATO), which can be further utilized as the templates to prepare various confined or patterned nanostructures [5], including quantum dots [6], nanowires/nanotubes [5,7-9], and even nanonets [8,10-12]. This process possesses an advantage that the key structural parameters of the porous membranes (pore diameter, inter-pore distance, and membrane thickness) can be tuned by carefully controlling the anodization conditions. Porous ATO has drawn particular attention due to the significant role of TiO_2 _in DSSCs [13,14], photocatalysis [15], water photoelectrolysis [13], and organic pollutants degradation [16]. So far, most TNT arrays have been prepared on flat Ti foils [17] as well as other flat substrates such as glass, alumina, and silicon [18]. Wang and co-workers [19] recently reported the fabrication of a DNA-like photo-electrode via electrochemical anodization as well as the application of this photo-electrode in liquid DSSCs. Another group of scientists [20] fabricated the liquid DSSCs by employing the TNT arrays. The device structure by inserting the photo-anode in a capillary glass tube along with a platinum wire as the counter electrode, however, limited the device's flexibility and thus restricted post-processing of solid solar cells.

Here we report the electrochemical anodization of a thin Ti wire into a cylindrical core-shell-like TNT array that can directly serve as a DSSC anode. This anode structure wrapped by a twisted counter electrode can be feasibly devised into a semi- or all-solid DSSC, and its performance has been improved remarkably. Moreover, a detailed study of TNT array structures and their charge-transfer capacities with respect to those of the anodes based on TiO_2 _nanoparticles was carried out, providing some insights into the performance optimization of the devised DSSCs.

## Experimental

### Anodization of Ti wires

Anodization of a thin Ti wire into a cylindrical core-shell-like TNT array is quite simple and straightforward. The whole process consists of three essential steps: (i) electropolishing Ti wires; (ii) anodizing the electropolished Ti wires; and (iii) devising the anodized Ti wires into DSSCs. A thin Ti wire (Sigma-Aldrich; 127-250 μm in diameter, 99.7% purity) was first washed with isopropanol in an ultrasonic bath and subsequently anodized in a mixed electrolyte of C_2_H_5_OH (700 ml/l), isopropanol (300 ml/l), AlCl_3 _(60 g/l), and ZnCl_2 _(250 g/l) [21]. The electropolishing was carried out at 90 V and 25°C for 10 s by using a Pt foil as the counter electrode. The anodization was conducted at 60 V in ethylene glycol containing 0.25 wt% NH_4_F. The anodized Ti wire was then immersed into a mixture of Br_2 _and CH_3_OH (1:10 vol%) for 5-10 h to dissolve the Ti core, leading to a free-standing and cylindrically tubular TNT array which structure was characterized by field emission scanning electron microscopy (FESEM, Hitachi S4800 and FEI Quanta 200F), transmission electron microscopy (TEM, JEOL JEM-200CX), and X-ray diffraction (XRD, Rigaku D/MAX-200). In addition, the nanoporous layer composed of 20 μm TiO_2 _particles was produced by P25 colloid coating and subsequently sintering at 450°C.

#### Assembly of DSSCs

The F-DSSCs were fabricated by directly employing the prepared and annealed cylindrical core-shell-like TNT array as the working electrode with its inner Ti core as the electric conduction lead. Two types of F-DSSCs were produced, i.e., semi- and all-solid F-DSSCs. Specifically, the anodized Ti wire was first sensitized by 3 × 10^-4 ^M N3 dye [*cis*-*bis*(isothiocyanato) *bis*(2,2"-bipyridyl-4,4"-dicarboxylato)-ruthenium(II)] for 12 h. Then, the semi-solid F-DSSC with a structure of Ti/TNTs/N3/electrolyte/Pt (0.05 mm, 99.9%) (see context described later) was assembled by adopting a similar method reported previously [2]. Particularly, to improve the stability and reproducibility of the cell, a gel of poly(ethylene glycol) (ca. 8000 Da, Aldrich, St. Louis. MO) (0.2 g/ml) + 0.5 M LiI (Aldrich, St. Louis. MO) + 0.05 M I_2 _(AR) + 3-methyl-2-oxazolidinone (Aldrich, St. Louis MO)/CH_3_CN (1:9) was employed as the electrolyte. The all-solid F-DSSC with a structure of Ti/TNTs/N3/CuI/Au (0.03 mm, 99.9%) was fabricated by a method reported in the literature [22].

#### Measurements of the DSSC performance

The light beam with an intensity of 100 mW cm^-2 ^was generated by YSS-50A (Yamashita DENSO, Tokyo, Japan). To exclude the efficiency improvement due to light bent or ambient light, the testing environment was carefully examined. The filling factor (FF) and overall conversion efficiency (*η*) were calculated as follows: FF = (*I*_opt _× *V*_opt_)/(*I*_sc _× *V*_oc_), *η = *(*I*_opt _× *V*_opt_)/*P*_in_, where *I*_opt _and *V*_opt _are the current and voltage at the maximum output power point, respectively. *I*_sc _and *V*_oc _are the short-circuit current and open-circuit voltage, respectively. *P*_in _is 100 mW cm^-2 ^here. Impedance spectral measurements were performed under the sunlight with a ZAHNER Elektrik IM6e impedance measurement unit using 20 μm TiO_2 _film samples.

## Results and discussion

### Morphology and structure characterization of TNT array

**Figure 1:**
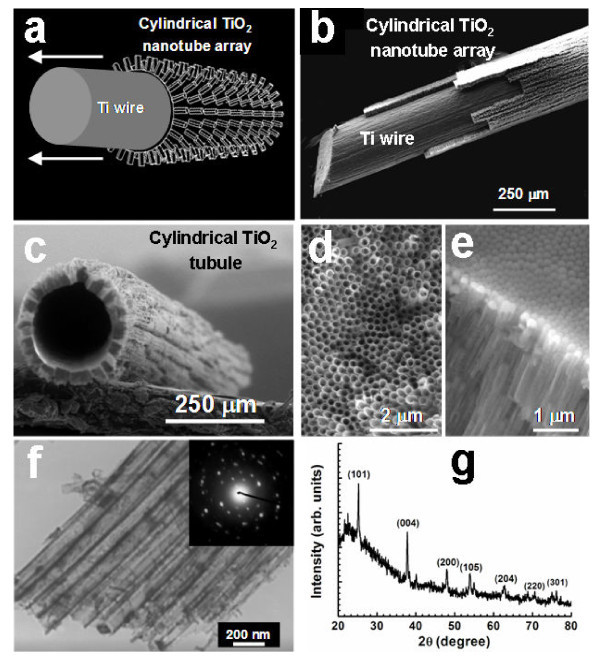
**Structure characterization of the TNT arrays (a) Schematic diagram of an anodized Ti wire with a TNT array outer layer wrapping the inner Ti core**. **(b) **FESEM image of an anodized Ti wire which outer TNT array was partially peeled off. **(c) **FESEM image of a free-standing cylindrical TiO_2 _tubule with its Ti core completely removed by chemical etching. Top **(d) **and bottom **(e) **view of the TNT array by FESEM. **(f) **TEM image of annealed TNTs. Inset: SAED of the TNTs. **(g) **XRD spectrum of the annealed TNT array.

The structures of the hierarchical crystalline TiO_2 _array are shown in Figure 1. A schematic drawing of an anodized Ti wire consisting of an inner Ti core and a TNT array outer layer is shown in Figure 1a, which was further confirmed by the FESEM image (Figure 1b). After the Ti core being completely etched off, a free-standing and cylindrical TNT tube survived, as shown in Figure 1c, which outer diameter, tube thickness, and length were about 250 μm, 40 μm, and 5-10 cm, respectively. The top (Figure 1d) and bottom (Figure 1e) views of a piece of TNT array peeled off from the anodized Ti wire (Figure 1b) confirmed the existence of the TNT array surrounding the Ti wire. It is apparent that the top layer consisted of open-ended TNTs (Figure 1d) while the underlying layer consisted of a continuous TiO_2 _barrier layer (Figure 1e) that tightly held the TNTs and inner Ti core together. A closer examination by TEM (Figure 1f) revealed that the diameter and wall thickness of TNTs were around 175 and 35 nm, respectively. Systematic experiments (not shown here) indicated that the TNT diameter could be fine-tuned by changing the anodization voltage. The as-prepared TNTs were amorphous in nature, which can, however, be transformed into a polycrystalline anatase structure by thermal treatment at 450°C, as shown by the selected-area electron diffraction (SAED, inset in Figure 1f) as well as the XRD pattern (Figure 1g) of the annealed TNTs. All these results evidenced that a cylindrical tubular TNT array was successfully prepared.

**Figure 2:**
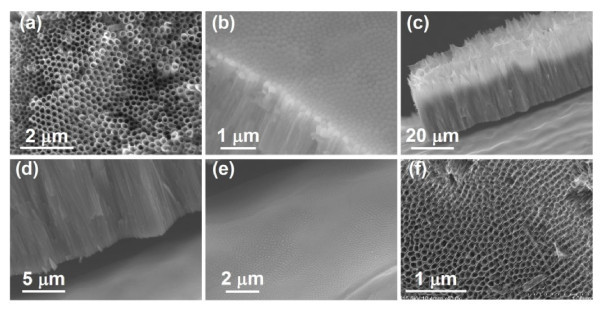
**Large-scale FESEM images **of the top (**a**) and back (**b**) sides of the TNTs array on the Ti wire; (**c**) large-scale and (**d**) enlarged FESEM image of the TNTs array being lifted off from the underlying surface; (**e**) FESEM image of the underlying bumpy surface of an inner Ti wire core; (**f**) FESEM image of the TNTs prepared by anodization of the Ti wire at 30 V in an electrolyte of ethylene glycol containing 0.20 wt% NH_4_F.

Furthermore, the structural parameters of the prepared TNTs can be tuned by varying the anodization conditions including the electrolyte type, the anodization voltage, and the anodization time. TNTs existed in the outer layer of the Ti wire after anodization, subsequent chemical etching, and ultrasonication treatment. The outer (Figure 2a) and inner (Figure 2b) sides of the anodized layer were open-ended TNTs and the TiO_2 _barrier layer, respectively. The anodized layer can be peeled off from the Ti substrate via vigorous ultrasonication treatment (Figure 2c,d), while the exposed surface of the Ti wire substrate became bumpy and roughened. The pattern of the bumps well matched with that at the inner side of the TNTs (Figure 2b). These results suggested that TNTs were indeed formed and connected to the un-anodized Ti core through the connecting TiO_2 _barrier layer. By varying the electrolyte concentration and anodization voltage, from 0.25% NH_4_F and 60 V to 0.2% NH_4_F and 40 V, the TNT diameter can be down-sized from 175 nm (Figure 2a) to 100 nm (Figure 2f).

**Figure 3:**
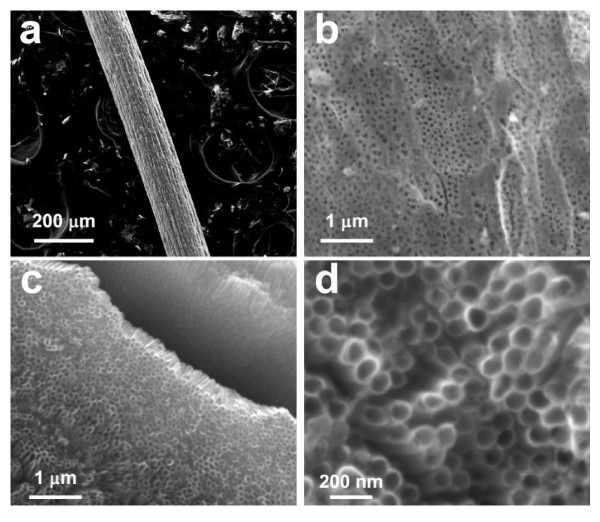
**Characterization of surface topography (a) FESEM image of an anodized Ti wire with a diameter of 127 μm; (b) FESEM image of outer surface of an as-anodized Ti wire**. **(c) **Large-scale FESEM image of an anodized Ti wire after chemical etching or ultrasonication. All exposed TiO_2 _nanotubes were open-ended and the broken part in the porous ATO membrane shows clearly the side-view of the nanotubes. **(d) **Enlarged FESEM image of individual TiO_2 _nanotubes which average diameter is about 105 nm.

By employing the similar anodization process, one can readily anodize thinner Ti wires such as a 127 μm Ti wire into an anode for DSSCs. The as-anodized Ti wire (Figure 3a) was covered by an outer layer of porous membrane that was actually cylindrical tubular TNTs. This porous layer (Figure 3b) could be easily removed by ultrasonication, leading to the smooth and open-ended TNTs (Figure 3c) which length could be tuned by varying the anodization duration time. A closer look at the structure with FESEM revealed that the produced TNTs were about 105 nm in diameter at an anodization voltage of 30 V (Figure 3d), and were held to the un-anodized Ti core by the TiO_2 _barrier layer in between. Both the amorphous TNTs and underlying TiO_2 _barrier layer turned into polycrystalline anatase after being annealed at 450°C. The cylindrically core-shell-like TNT array of various structural parameters, including the Ti wire length and diameter as well as the TNTs' diameter, length, and wall thickness, could be prepared by controlling the electrochemical anodization parameters.

### Photovoltaic performance of the devised DSSCs

**Figure 4:**
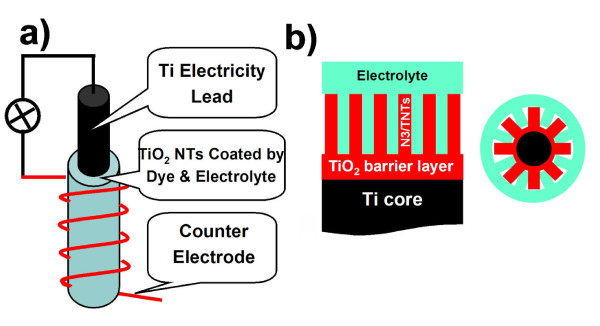
**Schematic diagram of (a) F-DSSC device**. **(b) **Illustrative axial (half) and radial cross sections of the TNT/Ti wire coated by dye/electrolyte.

**Figure 5:**
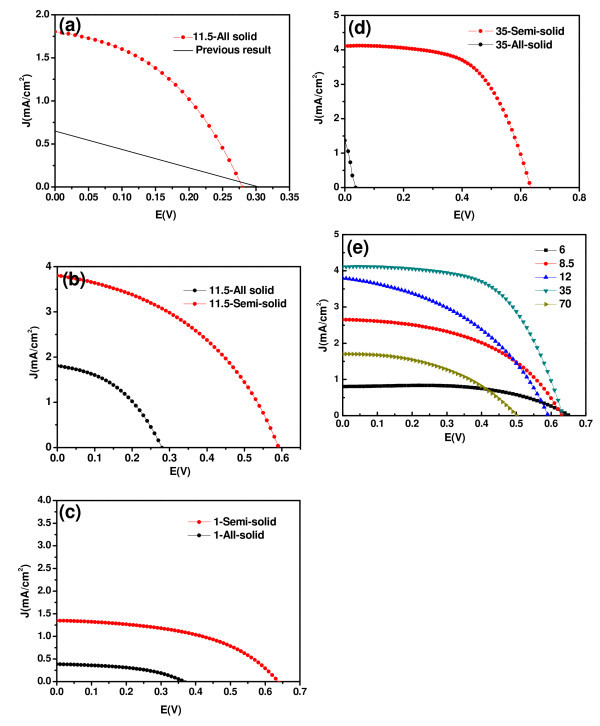
**Experimental evaluations of the F-DSSC performances**. **(a) **Measured current density versus voltage curves for the all-solid F-DSSC (as a function of the TNT length). The straight lines are adapted from references [22]. **(b-d) **Comparisons of the current density versus voltage curves between semi- and all-solid F-DSSCs of different TNT lengths in the anodized and annealed Ti wires. Terminology: 1-semi-solid means the semi-solid F-DSSC fabricated from the TNTs which average length (or TNT layer thickness) is 1 μm, 35-all-solid means the all-solid F-DSSC fabricated from the TNTs which average length (or TNT layer thickness) is 35 μm, and so on. Average TNT length or TNT layer thickness: (b) 1 μm; (c) 11.5 μm; and (d) 35 μm. **(e) **Experimentally measured current density versus voltage curves for the semi-solid F-DSSCs as a function of the TNT length.

Both semi- and all-solid F-DSSCs were assembled by using the as-prepared cylindrical core-shell-like TNTs arrays as the anodes (Figure 4a,b). The performances of both F-DSSCs were measured as a function of the TNT layer thickness (i.e., the length of the TNTs inside), as shown in Figure 5. An optimized performance was achieved with the TNT layer of 35 μm in thickness for the semi-solid DSSCs, as shown by Figure 5e. This is about nine times thicker than that previously reported [2]. Compared with previous results, the *I*_sc _(short-circuit current) increased by a factor of 3.5 (from 1.3 to 4.2 mA cm^-2^) while the FF reached 0.59 from 0.38. The *E*_oc _(open-circuit voltage) was 0.63 V. The light conversion efficiency, η, calculated by using the projection area as the light illumination area was about 1.5%, which is quite competitive to the previous results.

The all-solid F-DSSC was devised by using the TNT array which diameter and length were 105 nm and 11.5 μm, respectively. This TNT array was achieved by anodizing the Ti wire of 250 μm in diameter. CuI was used as the solid electrolyte. In comparison with previous results [22], the value *I*_sc _was increased by twofolds (from 0.63 to 1.80 mA cm^-2^) while its *E*_oc _retained around 0.3 V. The experimentally measured FF value of this all-solid F-DSSC (about 0.43) was nearly twice as large as the literature data (0.23). Its η also significantly increased from below 0.06% to about 0.21%, as shown in Figure 5a.

**Figure 6:**
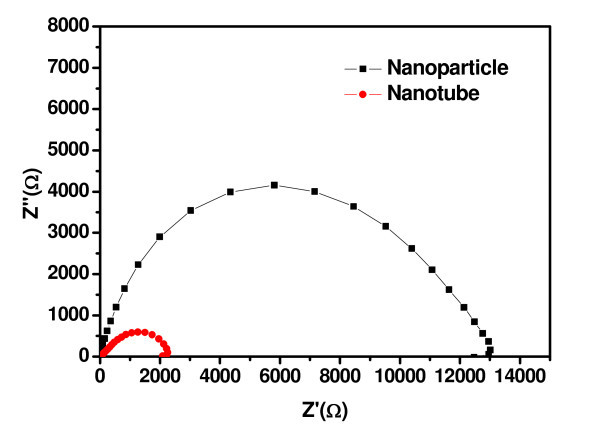
**Impedance spectra of the electrodes fabricated from TiO**_**2 **_**nanotube and nanoparticle films of 20 μm in thickness, measured in the sunlight without applied bias.**

According to the results depicted in Figure 5b,c,d,e, several experimental observations were noticed: (a) the performances of both semi-solid and all-solid DSSCs changed drastically with the TNT layer thickness. (b) The performances of all-solid F-DSSCs were always lower than those of the semi-solid F-DSSCs of the same TNT layer thickness. (c) The performances of all-solid F-DSSCs deteriorated much faster than those of the semi-solid F-DSSCs of the similar TNT layer thickness. The substantial performance enhancement observed for both F-DSSCs suggested that the poly-crystallized TNT arrays in the anodized Ti wires better the carrier-transfer in the devised DSSCs. This was further supported by impedance measurements. Two devices with the Ti/TiO_2_/CuI/Au structure were fabricated by using the anodes consisting of either TiO_2 _nanoparticles or TNTs prepared by coating or anodization method. The impedance of the Ti/TNTs/CuI/Au DSSC was much smaller than that of the Ti/TiO_2 _nanoparticles/CuI/Au device (Figure 6), implying that the carrier-transfer in the TiO_2 _barrier layer improved remarkably. This remarkable difference between the impedances of both DSSCs suggests that the Ti/TNTs/CuI/Au structure may possess a much better carrier-transfer capability than the Ti/TiO_2 _nanoparticles/CuI/Au one. The performances of our F-DSSCs, either semi-solid or all-solid, based on the as-prepared cylindrical core-shell-like TNT array anodes, were much better than or at least competitive to those of conventional flat-type DSSCs on flexible substrates reported previously [1,2,23], although being still poor compared with traditional Grätzel DSSCs.

There are several possible reasons that may explain the performance enhancement of our F-DSSCs. First, the grain boundaries and surface defects are substantially suppressed in our ordered and polycrystalline TNT array anodes, opening more carrier-transfer channels, as evidenced by the impedance measurements. Comparing with the nanoparticles, nanotubes may contain less surface defects and grain boundaries. The existence of surface defects can increase the charge recombination probability, which in turn reduces the DSSCs' performance. One possible way to suppress the charge recombination is to improve the TNT surface morphology which accordingly reduces the surface defects. Ordered structures such as nanowires should contain a lower density of such surface defects, but suffer from the low surface to volume ratio that leads to much lower dye adsorption capability. Second, the projection areas of the DSSCs were simply taken as the illumination areas in our calculations. One might argue that the backside of the anode may also be illuminated due to light-scattering effect, which would accordingly contribute to the performance enhancement. However, the light scattering could also cause some loss of light illumination at the front side, which then actually trade off the possible light illumination enhancement at the backside of the anode. As a result, the total light absorption by the anode does not change very much. However, our experiment showed that the performance could be doubled by placing a mirror behind the F-DSSC, providing the direct evidence substantiating that the back light illumination did not seriously contribute to the performance of our F-DSSCs. Third, the hierarchical structure of our prepared TNT arrays could be a plus for the performance enhancement. It was previously reported that the nanotube structure was indeed in favor of light adsorption [24]. Presumably, micro-photon cages might be formed in the fiber-like TNT array anodes, which could obviously enhance the DSSC performance. It must be pointed out that the real dominating factor(s) responsible for the performance enhancement of our F-DSSCs is still elusive, and more experimental evidence should be collected before we can draw an unambiguous conclusion. A morphological change of the anode from plate to fiber not only alters the anode shape, but the surface curvature, the interfacial contact area, and the packing state along the surface normal direction as well. Previous reports [20] showed that the optimized anode thicknesses of the TiO_2 _nanoparticle and nanorod/nanowire films were about 10 and 4 μm, respectively [2]. However, the optimized thickness of our TNT anodes in this study was as large as 35 μm, remarkably different from that of the reported plate-type counterparts. The TNTs grown at the Ti fiber surface were perpendicular to the curved surface of the Ti fibers. Compared with the flat-type anodes, the TNTs at the outer side of our tabular TNT anode should be relatively less densely packed than those at the inner side (closer to the inner Ti core). Therefore, the outer side path for hole-transfer material is longer, and the contact between the dye-sensitized TiO_2 _and the hole-transfer material becomes better at the inner side. Such a structure should be helpful in improving the charge separation efficiency of the electrode and the electrolyte, suppressing the dark current [20] and the efficiency of carrier collection. This actually mimics the nutrition transport system of trees or human beings. However, if the thickness of the TNT layer becomes too thick, the performance of the F-DSSCs certainly worsens due to the limited mean free path of the carriers inside the TNTs.

ZnO is another widely used wide-band-gap semiconductor material in DSSCs, possessing physical properties similar to TiO_2_, but a higher electron mobility that would be favorable for electron transport. However, the instability of ZnO in acidic dye and the slow electron-injection kinetics from dye to ZnO prevent the ZnO-based DSSCs from achieving a higher conversion efficiency (the best efficiency reported up to date being only about 5.4%) than the TiO_2 _counterparts. For films containing ZnO nanofibers or nanotubes, a high electron mobility together with a low recombination rate should yield a much higher current than the ZnO nanoparticle films. However, the low surface areas of the nanowire/nanorod arrays seem to be a primary factor that limits the amount of dye adsorption and hence the conversion efficiency of the cells [25]. Wang and co-workers [26] reported a three-dimensional (3D) DSSC in which the ZnO nanowires grew perpendicular to the optical fiber surface, which could enhance the surface area for the interaction of light with the dye molecules. Its conversion efficiency was 3.3%, much higher than that based on a flat substrate surface (about 1.5% [27]). Although the efficiency of our TNT-based DSSCs was not as high as that ZnO-based 3D DSSCs at the moment, the facile fabrication, simply post-processing, and flexibility of the TNT fiber anode as well as the outstanding chemical and physical properties of TiO_2 _make us believe that their performance can be potentially improved with further optimizations of their structural parameters.

## Conclusions

We have successfully fabricated cylindrical core-shell-like TNT arrays through anodization of thin Ti wires. These flexible TNT arrays became polycrystalline after post-annealing at 450°C and could be woven into a variety of structures in which light might be hierarchically scattered and trapped. The structural parameters of both TNTs and Ti wires can be fine-tuned by varying the anodization parameters. The as-anodized Ti wires after annealing were directly used as anodes to devise semi-solid and all-solid fiber-type DSSCs. The twisting style of the counter electrode and working electrode did not impact the flexibility of the TNT array anode. Experimental evaluations showed that the *I*_sc _for both DSSCs increased at least by two times, and their FFs greatly improved compared to their nanoparticle counterparts. Particularly, the η of the semi-solid F-DSSC was above 1.5%, better than or competitive to that of other DSSCs fabricated by depositing disordered TiO_2 _particles on flexible flat or fiber substrates. However, the efficiency of the all-solid DSSC was still relatively low, i.e., about 0.21%, though much better than previously reported result. Further optimization of the F-DSSC performances is underway in our lab.

## Abbreviations

ATO: anodic aluminum oxide; F-DSSC: fiber-type dye-sensitized solar cells; FESEM: field emission scanning electron microscopy; FF: filling factor; ITO: indium tin oxides; SAED: selected-area electron diffraction; TEM: transmission electron microscopy; TNT: TiO_2 _nanotube; XRD: X-ray diffraction.

## Competing interests

The authors declare that they have no competing interests.

## Authors' contributions

JY, XT, CG, JL, YH and KW contributed to the fabrication of the TiO_2 _nanotube arrays; DW, XF and DZ contributed to the assenmbly of DSSCs and performance measuremenet. All authors read and approved the final manuscript.
